# Signal Detection Theoretic Estimates of the Murine Absolute Visual Threshold Are Independent of Decision Bias

**DOI:** 10.1523/ENEURO.0222-24.2024

**Published:** 2024-10-09

**Authors:** Sam LaMagna, Yumiko Umino, Eduardo Solessio

**Affiliations:** Center for Vision Research, Department of Ophthalmology and Visual Sciences, SUNY Upstate Medical University, Syracuse, New York 13210

**Keywords:** absolute visual thresholds, mouse vision, operant behavior, response bias, theory of signal detection, visual noise

## Abstract

Decision bias influences estimates of the absolute visual threshold. However, most psychophysical estimates of the murine absolute visual threshold have not taken bias into account. Here we developed a one-alternative forced choice (1AFC) assay to assess the decision bias of mice at the absolute visual threshold via the theory of signal detection and compared our approach with the more conventional high-threshold theoretic approach. In the 1AFC assay, mice of both sexes were trained to signal whether they detected a flash stimulus. We directly measured both hit and false alarm rates, which were used to estimate *d′*. Using the theory of signal detection, we obtained absolute thresholds by interpolating the intensity where *d′ *= 1 from *d′*-psychometric functions. This gave bias-independent estimates of the absolute visual threshold which ranged over sixfold, averaging ∼1 R* in 1,000 rods (*n* = 7 mice). To obtain high-threshold theoretic estimates of the absolute visual threshold from the same mice, we estimated threshold intensities from the frequency of seeing curves, corrected for guessing. This gave us thresholds that were strongly correlated with decision bias, ranging over 13-fold and averaged ∼1 R* in 2,500 rods. We conclude that the theory of signal detection uses false alarms to overcome decision bias and narrow the range of threshold estimates in mice, providing a powerful tool for understanding detection behavior near absolute visual threshold.

## Significance Statement

The detection of dim flashes of light depends both on the number of photons delivered by the flash and on the level of spontaneous neural activity, which observers can misclassify as incident photons. Such ambiguity introduces decision biases, which can make observers appear more, or less sensitive to light, increasing the range of sensitivity estimates. We found that the theory of signal detection provides a framework for dealing with these biases in mice by taking spontaneous neural activity into account, reducing the range of sensitivity estimates during the detection of dim, brief, flashes of light.

## Introduction

The vertebrate visual system displays immense sensitivity. The absolute visual threshold is the least amount of light required for sensing vision. In humans, estimates of the absolute visual threshold under optimal conditions range from a single rod absorbing a single photon to approximately a dozen rods absorbing single photons each (Hecht et al., 1942; van der Velden, 1946; Sakitt, 1972; Tinsley et al., 2016). This variation is due to an observer's tendency to respond independent of stimulus characteristics, that is, decision bias (Macmillan and Creelman, 2005). One framework for understanding decision bias is the theory of signal detection, which hypothesizes that bias reflects the placement of internal criteria that an observer uses to decide whether a stimulus is present or not (Green and Swets, 1966). Application of the theory of signal detection to the absolute visual threshold accounts for the effects of bias in human psychophysical studies (Barlow, 1956; Sakitt, 1972, 1974; Teich et al., 1982; Makous, 1990; Donner, 1992). As insightful as human psychophysical studies have been, their contribution to understanding the neural basis of vision at absolute threshold is limited, as the requisite experimental manipulation of underlying neural circuitry is not feasible in humans.

We use mice to study vision at absolute threshold. Mice and primates share many features including single photon detection by rods (Baylor et al., 1984; Doan et al., 2006) and the high convergence of rod pathways (Dunn et al., 2006) observed in the human peripheral retina (Lee et al., 2019). Indeed, studies of the murine retina have provided much of our understanding concerning rod-driven responses in dim lights (Deans et al., 2002; Völgyi et al., 2004; Dunn et al., 2006; Jin et al., 2015; Smeds et al., 2019). However, there are only a handful of psychophysical investigations of the murine absolute visual threshold (Hayes and Balkema, 1993a, b; Herreros De Tejada et al., 1997; Nathan et al., 2006; Naarendorp et al., 2010; Smeds et al., 2019). In mice, as in humans, there is substantial variation in the estimates of threshold intensities. However, previous studies have largely ignored the influence of response bias on measures of the murine absolute visual threshold. This is unfortunate, because response bias is not simply a nuisance, but holds valuable information about how perception works. For instance, proper measurement of response bias can provide deeper insight into how observers process signal and noise and how response times are related to perceptual judgments (Green and Swets, 1966; Luce, 1986).

We applied the theory of signal detection to understand the impact of response bias near absolute visual threshold in mice using a procedure known as a “yes/ no” or one-alternative forced choice (1AFC) assay (Kingdom and Prins, 2010). We will refer to it as a 1AFC assay throughout the rest of the paper. The strength of the 1AFC assay is that it provides direct measures of both the hit and false alarm rates, which when combined with the theory of signal detection can account for decision bias (Green and Swets, 1966; Macmillan and Creelman, 2005; Kingdom and Prins, 2010). Our findings show that absolute thresholds were strongly correlated with decision bias and that this bias was well accounted for by the theory of signal detection, suggesting that decision bias is a measurable factor in murine psychophysical tasks.

## Materials and Methods

We used a total of seven C57BL/6J mice (Jackson Laboratory). Mice (two male, five female) were maintained on a 14 h light/10 h dark cycle and dark-adapted for at least 12 h before each experiment. All tests were performed during subjective daytime hours, Zeitgeber time of 4–8 h. To maintain motivation for learning and performing the operant behavioral task, each mouse was kept on a food-restricted schedule (resulting in body weight between 80 and 90% of expected weight) and single housing as described by Umino et al. (2018). Food restriction began at 10–12 weeks of age, 2 weeks prior to starting training. At the time of the experiment, mice were ∼6 months of age. We also determined whether the animals were lethargic or showed any signs of dehydration. If the animals’ body weight dropped to <80% of their normal body weight, we supplemented their diet with additional dry food on a daily basis. We progressively increased the daily amount of dry food that the underweight mouse received, in 0.25 g increments, until the mouse reached the target of >80% expected weight. All procedures in this study were approved by the Institutional Animal Care and Use Committee at SUNY Upstate Medical University and conducted in accordance with the National Academy of Sciences’ *Guide for the Care and Use of Laboratory Animals* and in compliance with the Association for Research in Vision and Ophthalmology Statement for the Use of Animals in Ophthalmic and Vision Research.

### Operant behavioral assay to test absolute visual thresholds in mice

**Figure 1.**
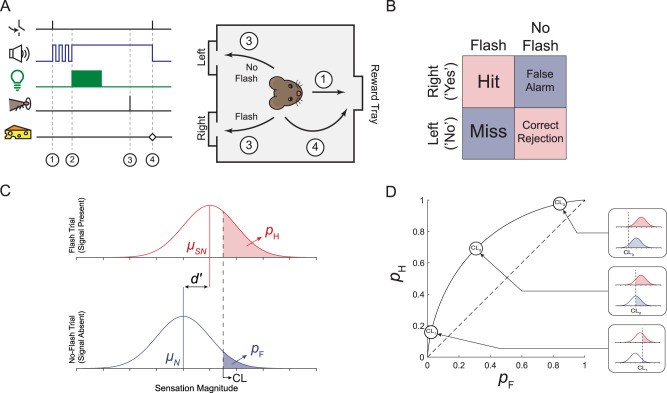
A one-alternative forced choice (1AFC) task for measuring sensitivity to flash stimuli. ***A***, Schematic of the 1AFC task. (1) Mice self-initiate trials by visiting the reward tray, which commences an auditory “countdown” cue. (2) At the end of the cue, a solid tone begins. During stimulus trials, this is coincident with the presentation of a flash stimulus. During catch trials, there is no flash stimulus given. (3) For stimulus trials, mice are trained to visit the right nose-poke. For no-flash trials, mice are trained to visit the left nose-poke. (4) Mice return to the reward tray; mice receive a reward only if they visit the correct nose-poke. ***B***, Decision matrix. For stimulus trials, correctly visiting the right nose-poke (RNP) is termed a hit; incorrectly visiting the left nose-poke is termed a miss. For no-flash trials, correctly visiting the LNP is termed a correct rejection (CR); incorrectly visiting the RNP is termed a false alarm (FA). ***C***, Definition of *d′*. According to the theory of signal detection, we can think of all perceptual judgments as discriminating signal from noise. In the absence of stimulus, basal neural activity will elicit sensations of a certain magnitude; we can describe the resultant sensation magnitudes by a probability distribution with mean *μ*_N_. By sensation magnitude, we mean the intensity of the sensation on a psychological scale. When given a stimulus, there will be an increase in neural activity that will shift this distribution to a new mean *μ*_SN_. This distance, which is the difference between means, is the *d′*. Since the distributions overlap, an observer in a detection task is liable to confuse sensations coming from basal neural noise as a true signal. Thus, the observer establishes an internal criterion location (CL), above which an observer will categorize as a signal and below which an observer will categorize as the absence of a signal. The *p*_H_ and *p*_F_, then, are simply the integral of the probability distribution from the criterion to +∞. ***D***, The isosensitivity curve. The placement of the observer's criterion is dynamic and reliant on the payoffs, punishments, and a priori probabilities of signal presentation during a detection task. Since the *p*_H_ and *p*_F_ are reliant on the criterion, they will both change as an observer shifts their criterion. At CL_1_, the observer is behaving very conservatively; they are biased toward saying “no” more often. At CL_3_, the observer is behaving very liberally; they are biased toward saying “yes” more often. At CL_2_, the observer is behaving in an unbiased manner. No matter the bias of the observer, however, the *p*_H_*–p*_F_ pairs all fall along the same isosensitivity curve. This isosensitivity curve reflects the *d′*, which remains constant as bias changes.

To determine absolute visual thresholds in mice, we utilized a modified operant conditioning assay (Umino et al., 2018). Tests were performed using a control and conditioning system (Lafayette Instruments) consisting of eight experimental chambers controlled by ABET II software (Lafayette Instruments). The user-defined program scheduled the tests, recorded the responses, and presented the reinforcements. Each chamber consisted of a reward tray and two equidistant nose-pokes located on the opposite side (Fig. 1*A*). Light stimulation was presented by a programmable LED (Luxeon) placed overhead. The LEDs had central emission at 496 nm and a half-maximal bandwidth of 28 nm as measured with a spectral radiometer (Photo Research, SpectraColorimeter, model PR-650). Flash intensities were controlled by pulse width modulation and neutral density filters. Light diffusers were used to keep luminance levels between all surfaces of the chamber (ceiling, walls, and floor) uniform (0.3 log-unit maximal difference) as measured with a Graseby S370 optometer equipped with a photometric filter and 15° Lumilens.

Mice initiated each 1AFC trial by visiting the reward tray (Fig. 1*A*, Step 1). Following the visit to the reward tray, an auditory “countdown” cue began, consisting of three 0.1 s high-frequency tones. Furthermore, 0.1 s after the last countdown tone, a solid tone was presented concurrently with the light stimulus (which can be a flash, or no flash, depending on the trial, Fig. 1*A*, Step 2). The solid tone terminated when the mouse responded or, in the absence of a response, after 14 s, when the trial was aborted (Fig. 1*A*, Step 4). A response was defined as the last nose-poke visit made by the mouse before it returned to the reward tray (Fig. 1*A*, Step 3). Correct responses, which include hits and correct rejections, were rewarded with a small amount of commercially available Ensure Nutrition Drink (5–10 μl, Abbott Laboratories), rewarding 100% of the hits and 100% of the correct rejections.

Following training (details below), we tested daily the ability of mice to detect flashes applied on a dark background. Prior to each testing session, mice were dark-adapted overnight for at least 12 h to optimize their visual sensitivity and were then handled under dim red illumination (<0.2 R*/rod/s at the position of the mouse). Each experimental session consisted of 400 trials following a warm-up session of at least 100 corrective trials. As shown in a study by Umino et al. (2018), mice are able to complete at least 400 trials of the 1AFC without showing any signs of fatigue. The duration of each session was 1.5–2 h. During the sessions, mice were continuously maintained in dark conditions; the light stimulus consisted of a brief, 0.06 s flash applied in the dark. A flash duration of 0.06 s falls well within the integration time of the mouse rod (Burns et al., 2002) and the human psychophysical critical duration (Barlow, 1958), the range of temporal linear summation. The only stimulus variable is the intensity of the flash.

Each completed trial has four possible outcomes: (1) correctly visiting the right nose-poke (RNP) on flash trials, i.e., a hit; (2) correctly visiting the left nose-poke on no-flash trials, i.e., a correct rejection (CR); (3) incorrectly visiting the right nose-poke on a no-flash trial, i.e., a false alarm (FA); and (4) incorrectly visiting the left nose-poke on a flash trial, i.e., a miss (Fig. 1B). For a given experimental session we determined the hit rate, or the probability of a hit (*p*_H_), and the FA rate, or the probability of a FA (*p*_F_). From the *p*_H_ and *p*_F,_ we then calculated the sensitivity index (*d′*):

$$d^{\prime }=Z(\;p_{H})-Z(\;p_{F})$$

where Z(·) is the inverse of the standard normal distribution and *d′* is a measure of visual sensitivity that operates independently of observer bias (see Fig. 1*C,D* and respective legends for description; Green and Swets, 1966; Macmillan and Creelman, 2005).

### Conditioning and training schedules

**Figure 2.**
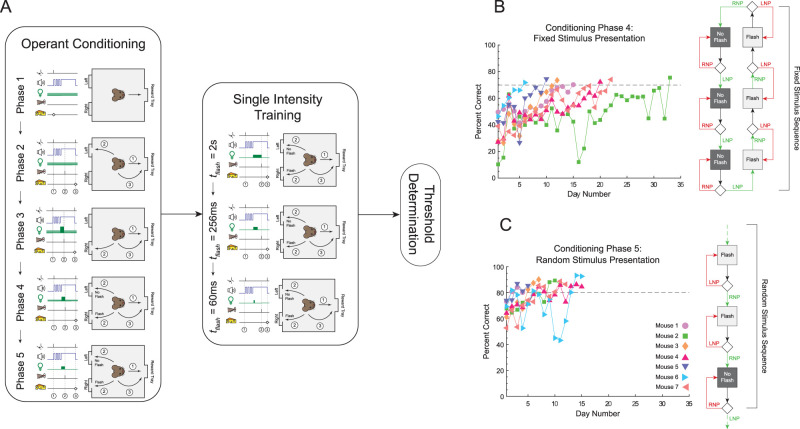
Conditioning and training progression. ***A***, An outline of the conditioning and training regimen used to teach and stabilize mouse performance, respectively (see Materials and Methods). ***B***, Progression of training for Phase 4. Fraction correct of training Phase 4 plotted as a function of training day number. Data are shown for each individual mouse, denoted by color code. The dotted reference line is 0.7, the fraction correct mice must exceed to finish training Phase 4. ***C***, Progression of training for Phase 5. Fraction correct of training Phase 5 plotted as a function of training day number. Each mouse is color-coded as in ***B***. The dotted line is 0.8, the fraction correct mice must exceed for 3 consecutive days to finish training Phase 5.

Mice first learned the 1AFC task via a five-phase shaping protocol (Fig. 2*A*). The conditioning flashes were relatively long (2 s duration) because our pilot studies demonstrated that long conditioning flashes are more effective than short ones in shaping behavioral responses. Following the completion of the conditioning protocol, mice began a single-intensity training protocol designed to stabilize responses to the short, 0.06 s flashes used later in the multi-intensity experiments. We conditioned and trained mice such that “yes” responses (i.e., hits and false alarms) corresponded to right nose-pokes and that “no” responses (i.e., correct rejections and misses) corresponded to left nose-pokes. As a control for whether this configuration may bias mouse responses, we trained and tested one mouse (Mouse 6) with a reversed nose-poke–response relationship. That is, the left nose-poke was associated with “yes” responses, and the right was associated with “no” responses. All other aspects of training and testing were identical to the other mice tested. Mouse 6 displayed similar behavior as the other mice tested, despite the reordering of the configuration. The conditioning and training schedules were as follows.

### Operant conditioning schedule

#### Phase 1: reward tray conditioning

The purpose of this initial phase was to teach mice to associate visiting the reward tray with the onset of the auditory cue and receiving the reward. Every visit to the reward tray resulted in the onset of the auditory cue and the dispensing of the reward. The chamber was illuminated with a steady background light eliciting ∼300 R*/rod/s. To direct mice to visit the reward tray, both nose-pokes were covered so the mouse did not have access to them (Fig. 2*A*). Phase 1 was completed when a mouse visited the reward tray at least 300 times during a training session (typically ∼2–3 h). It took 1–2 d to complete Phase 1.

#### Phase 2: tone-only nose-poke conditioning

The purpose of Phase 2 was to teach mice to visit the left nose-poke when the auditory cue was presented (Fig. 2*A*). Mice only had access to the left nose-poke and reward tray in the illuminated chamber. Mice self-initiated trials by first visiting the reward tray, whereupon the auditory countdown and solid tone were given. To receive the reward, mice had to visit the nose-poke before returning to the reward tray. If a mouse took >40 s to complete this sequence, the trial was aborted. Phase 2 was completed when a mouse successfully completed at least 300 trials within a training session. It took an average of 7.1 d to complete Phase 2.

#### Phase 3: tone and flash nose-poke conditioning

The purpose of Phase 3 was to teach mice to visit the right nose-poke when a flash stimulus was presented with the auditory cue. Mice only had access to the right nose-poke and reward tray in the illuminated chamber. Mice self-initiated trials by visiting the reward tray, and the countdown cue was then given. Coincident with the transition to the solid auditory tone, an ∼3,000 R*/rod/s flash stimulus, 2 s in duration, was presented. To receive the reward, mice had to visit the nose-poke before returning to the reward tray. Like Phase 2, if >40 s elapsed after trial initiation, the trial was aborted (Fig. 2*A*). Phase 3 was completed when a mouse successfully completed 200–300 trials within an hour. It took an average of 3.3 d to complete Phase 3.

#### Phase 4: fixed stimulus presentation

The purpose of training Phase 4 was to teach mice to visit the left nose-poke during no-flash trials and the right nose-poke during flash trials. Mice had access to both nose-pokes and the reward tray. To accustom mice to operate under dimmer conditions, the steady illumination of the chamber was attenuated to ∼150 R*/rod/s, and the flash stimulus was attenuated to ∼1,500 R*/rod/s. Mice were presented with alternating blocks of three consecutive no-flash trials and three consecutive flash trials (Fig. 2*A*). Reward was only given when a correct choice was made (i.e., a hit or correct rejection). When an incorrect choice was made (i.e., an FA or miss), the same trial was repeated until a correct choice was made (Fig. 2*B*). We denoted these repeat trials as “corrective trials”. The time allotted per trial was lowered to 20 s. Phase 4 was completed when the percentage of correct trials exceeded 70%. This was the most time-consuming of the conditioning phases and took an average of 16.6 d to complete.

#### Phase 5: random stimulus presentation

The purpose of Phase 5 was to teach the visual detection task. The background illumination was removed, and the flash stimulus was further attenuated to 400 R*/rod/s. Flash and no-flash trials were presented randomly such that there was an approximately equal number of flash and no-flash trials (Fig. 2*A*). Phase 5 was completed when the percentage of correct trials exceeded 80% for 3 consecutive training days (Fig. 2*C*). Further, the time allowed per trial was lowered to 15 s. It took an average of 9.3 d to complete Phase 5 and a total of 37.6 ± 8 d to complete the entire conditioning schedule.

### Single-intensity training

Following completion of the conditioning protocol, mice began a three-phase single-intensity training protocol. The purpose of the single-intensity training was twofold: to stabilize performance without corrective trials and to slowly reduce flash duration from 2 s to our target flash duration of 0.06 s. Here, conditions were identical to conditioning Phase 5 (an ∼400 R*/rod/s stimulus was used with no steady background illumination), except that no corrective trials were given. Since no corrective trials were given, we used the *d′* to measure performance. Each training session consisted of 400 trials.

Prior to each training session, a “warm-up” session with corrective trials was completed. The stimulus parameters of the warm-up were identical to the subsequent training session. The warm-up session also acted as a means to check if a mouse was “one-sided” (i.e., responding repeatedly to one side without regard for the stimulus). If a mouse was one-sided, the probability of a flash trial was changed to heavily bias the opposite side to correct the behavior. Warm-up sessions typically lasted 30 min–1 h and consisted of at least 100 trials.

**Figure 3.**
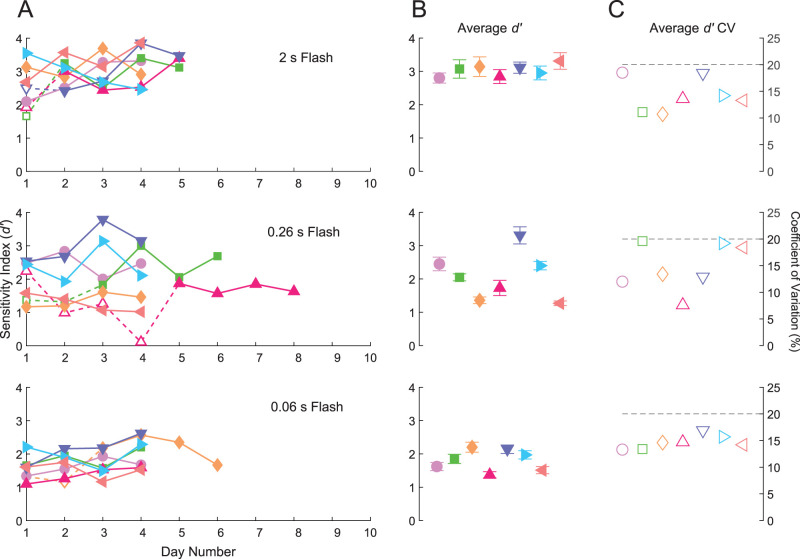
Single-intensity training progression. ***A***, Progression of single-intensity training over time. The sensitivity index (*d′*) plotted as a function of training day number for 2, 0.26, and 0.06 s, respectively. The filled symbols connected by solid lines indicate the final 4 training days used to determine average *d′* and coefficient of variation for stability. Empty symbols connected by the dashed lines indicate training sessions prior to stability. Colors and symbols correspond to the mouse ID's in Figure 2*C*. ***B***, Average *d′* values of the final 4 training days of the corresponding plots in ***A***; error bars are standard deviations. ***C***, Coefficients of variation for the final 4 training days. The dotted line is 20%; to finish single-intensity training for a given duration, CV ≤ 20%.

A phase was completed when the coefficient of variation (CV) for *d′* over 4 consecutive days was <20%. During Phase 1, mice responded to a 2 s flash stimulus. It took an average of 4.5 d to complete this phase (Fig. 3*A*). The average *d′* for a 2 s flash was 3.1 (Fig. 3*B*). During Phase 2, mice responded to a 0.26 s flash. It took an average of 5.3 d to complete this phase (Fig. 3*A*). The average *d′* for a 0.26 s flash was 2.0 (Fig. 3*B*). During Phase 3, mice responded to a 0.06 s flash. During this phase, mice were dark-adapted overnight preceding training. It took an average of 4.3 d to complete this phase (Fig. 3*A*). The average *d′* for a 0.06 s flash was 1.8 (Fig. 3*B*). We did not enforce any minimal *d′* value, because our interest was in the day-to-day stability of performance. In all, it took an average of 14.2 d to complete the entire single-intensity training protocol.

### Measuring absolute thresholds

#### Multi-intensity experiments to determine absolute visual threshold

To measure absolute visual threshold, mice completed a series of multi-intensity experimental sessions. Each session consisted of 400 trials of four randomly presented intensity levels over a 1.7–2.0 log-unit range, with randomly interspersed no-flash trials. Each experimental session consisted of approximately equal numbers of flash and no-flash trials. The experimental session follows a warm-up session, as outlined above (see above, Single-intensity training). During the warm-up session, the flash duration was 0.06 s, and the flash stimulus intensity was chosen to be in the middle of the range presented during the experimental session.

#### Stimuli are progressively dimmed to facilitate detection at absolute visual threshold

**Figure 4.**
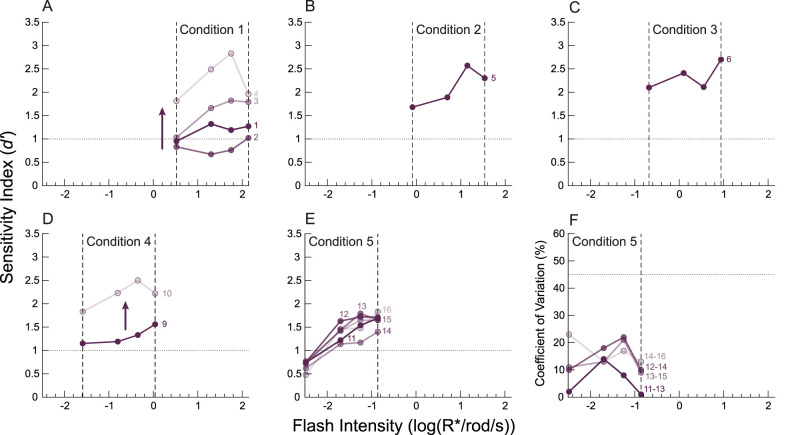
Multi-intensity experiment performance progresses over time. See Materials and Methods for a full description of performance progression. More transparent symbols/lines correspond to performance at later training days in a respective condition. The dotted horizontal lines show threshold performance (*d = *1). The dashed vertical lines delineate the intensity condition range. ***A***, Performance over 4 consecutive training days under intensity Condition 1. As time progresses, performance enhances. ***B***, Performance for a single day under intensity Condition 2. Because performance was so high, the mouse moved on to Condition 3. ***C***, Performance for a single day under intensity Condition 3. Again, because performance was so high, the mouse moved on to Condition 4. ***D***, Performance for 2 d under intensity Condition 4. Note that Days 7 and 8 were not shown because the mouse was unable to reliably respond on those days because we made the stimulus condition too dim, too quickly (see Materials and Methods). *d′* values increased substantially from Day 9 to Day 10. ***E***, Performance for 6 d under the final Condition 5. Under this condition, the mouse met our stability criteria (see Materials and Methods). ***F***, Demonstration of stable coefficients of variation (CV) under Condition 5. The CV values are obtained from moving averages and standard deviations of *d′* for each intensity, with a window of 3 d. Note that once our stability criteria have been met (*d′* values have a CV <45%, dashed line), they remain so for the whole 6 d.

Following the completion of the 0.06 s stimulus training, mice began to complete experimental sessions with relatively bright flash stimuli (∼1–100 R*/rod/s). The intensity was then gradually lowered by 0.6 log units, but attenuation could be more finely tuned by 0.3 log units if a mouse required a more gradual transition. This continued until a mouse's session-to-session performance stabilized near threshold. Pilot studies indicated that performance tended to stabilize when the CV for individual *d′* values is ≤45%. Because threshold is defined as the light intensity where *d′ = *1, our criterion for operating near absolute visual threshold was that the *d′* data systematically crossed *d′ = *1. By ensuring that performance for lower intensities was subthreshold, we interpolated the light intensity where *d′ = *1, minimizing error in our estimations. An example of this process is given in Figure 4.

#### Extending the psychometric functions to high intensities and reducing potential desensitization in the response to dim flashes

A goal of our study was to determine performance in response to dim flashes, as well as to analyze the asymptotic behavior that psychometric functions exhibit with brighter flashes. To decrease the possibility that presentation of a bright flash may desensitize the response to a subsequent dim flash, we ran two experimental sessions per day. In the first session, we tested the performance of dark-adapted mice to the four dimmest dim flashes. The second session followed immediately at the conclusion of the first session and tested the responses to bright flashes, which ranged from 1.2 to 1.5 log units brighter than the dim flashes of the first subsession, ensuring a partial overlap between the two intensity ranges (Fig. 6).

### Psychometric function fitting and threshold estimation

#### Theory of signal detection approach

The data that met the above criteria were used to fit a *d′*-psychometric function that relates stimulus intensity to *d′*. We found that our data were well fit with log-linear *d′*-psychometric functions, parameterized as follows:

$$d^{\prime }(I_{f})=\beta (\log\;(I_{f})-\log\;(I_{T}))+1$$

where *I*_f_ is flash intensity, *β* is the psychometric function slope, and *I*_T_ is the threshold intensity, i.e., the intensity where *d′ = *1. Psychometric functions were fit with nonlinear least squares regression.

#### High-threshold theory approach

The theory of signal detection holds that response bias reflects an observer's placement of a response criterion (Green and Swets, 1966). A competing theory of perception, the high-threshold theory, postulates that bias reflects random guessing (Blackwell, 1963; Green and Swets, 1966). Consequently, high-threshold theory predicts that one may eliminate response bias by simply correcting for this guessing. This is accomplished via the following expression (Pelli, 1985):

$$p_{C}=1-\frac{1-p_{H}}{1-p_{F}}$$

where *p*_C_ is the hit rate corrected for guessing. We estimated thresholds under high-threshold theory via the following psychometric function (Wichmann and Hill, 2001; Kingdom and Prins, 2010):

$$p_{C}(I_{f})=p_{F}+(1-p_{F}-p_{L})\Phi (\log\;(I_{f});\mu ,\sigma )$$

where *p*_L_ is the lapse rate; Φ is the cumulative normal distribution with a mean *μ*, which is defined as threshold; and *σ* is the standard deviation, where *σ*^−1^ is proportional to the slope of the function (Kingdom and Prins, 2010). Since Equation 4 corrects for guessing, we set *p*_F_* = *0 in Equation 4.

The lapse rate, *p*_L_, is the proportion of incorrect responses that are independent of stimulus intensity (Prins, 2012). For estimating the lapse rate, we examined the responses at intensities that are 1.2 log units higher than the intensities used for estimating threshold. At these levels, performance approaches asymptotes; we denote these intensities as asymptotic performance levels (APLs; Prins, 2012). Thus, to estimate high-threshold theoretic psychometric functions, we fit a model where:

$$p_{C}(I_{f})=1-p_{L}$$

when *I_f_  *═ APL, and Equation 4 otherwise (Prins, 2012). Psychometric functions were fit using nonlinear least squares regression.

### Single-intensity experiments

A subset of mice completed an additional set of single-intensity experiments to investigate how bias influences detection behavior. Mice were dark-adapted overnight and, after a warm-up session (see above), mice performed the 1AFC task with a flash intensity of either 1 × 10^−3^ R*/rod or 1 × 10^−2^ R*/rod. For each intensity condition, mice completed three experimental sessions, each consisting of 400 trials.

### Retinal irradiance

Retinal irradiance depends on the level of illumination at the cornea and the amount of light reaching the retina, determined by the area of the pupil. Luminance from incident from the chamber walls and ceiling was measured with a Graseby S370 optometer equipped with a 15° Lumilens. We found that the luminance of the surfaces was within approximately 0.3 log units of one another, consistent with an isoluminant visual environment. To determine retinal irradiance, we first made radiometric readings using the Graseby S370 in radiometric mode and by positioning the photodiode in the location of the mouse cornea directed at the back of the chamber, the nose-poke-side of the chamber, and ceiling of the chamber. These values were averaged together to arrive at the typical irradiance within the chamber. Radiometric measurements were converted to *Q*, photon flux density at each cornea (in photons/μm^2^/s), and then to the average number of photoisomerizations (R*) absorbed per rod per second in each retina using the expression derived by Lyubarsky et al. (2004):

$$R^{*}/\mathrm{rod}/s=Q(\lambda )\tau (\lambda )\frac{S_{\mathrm{pupil}}}{S_{\mathrm{retina}}}a_{C}(\lambda )$$

Here, “*λ*” is the stimulus wavelength (500 nm); “*τ*” (0.7) is the fraction of photons not absorbed by the ocular media; “*S*_pupil_” (4 mm^2^) and “*S*_retina_” (18 mm^2^) are the areas of the dark-adapted pupil and retina, respectively; and “*a*_C_” (0.87 μm^2^) is the end-on collecting area of a rod. We used the same values as Lyubarsky et al. (2004) for each variable except for pupil size. Because estimates of the dark-adapted pupil size of the freely behaving mouse range from 3.5–4.5 mm^2^, we set the *S*_pupil _*= *4 mm^2^ (Bushnell et al., 2017). Thus, 1 R*/rod/s corresponds to ∼7 photons/μm^2^/s at the cornea.

### Statistics and analysis

Unless otherwise specified, all statistical analyses were carried out using SAS software. For all statistical tests, *α* = 0.05. As outlined above, threshold intensity is estimated via the *d′*-psychometric function (Eq. 2). Equation 2 was fit to each mouse's individual *d′* data via nonlinear least squares regression, using the NLIN procedure (SAS Institute Inc., 2023). We also used nonlinear least squares regression to fit high-threshold theoretic psychometric functions (Eqs. 4, 5), via the NLIN procedure. We determined standard error estimates of all psychometric function parameters via nonparametric bootstrapping (Wichmann and Hill, 2001; Kingdom and Prins, 2010). Here, individual experiments are simulated by randomly sampling a binomial distribution specified by the *p*_H_ or *p*_F_ and the number of flash or no-flash trials, respectively. Simulated *p*_H_ and *p*_F_ were then used to calculate simulated *d′* values, from which we obtained simulated thresholds. The advantage of the nonparametric approach is that it requires no additional assumptions than what is used in calculating the *d′* and *p*_C_ [i.e., trials are independent of one another, there are only two possible responses, and that for each intensity, or no-flash trial, the probabilities of a hit, or FA, are constant from trial-to-trial (Wichmann and Hill, 2001)]. Simulated data were also used to construct bias-corrected and accelerated (BCa) confidence intervals (Efron and Tibshirani, 1994). For bootstrapping procedures, 1,000 iterations were used. For correlation analysis, we used the CORR procedure (SAS Institute Inc., 2023) to determine Pearson's correlation coefficients and associated *p*-values.

## Results

### Mice respond to brief flashes of light in an alternative forced choice assay

Mice were taught to respond to brief flashes of light applied on a dark background, using a one-alternative forced choice assay (Fig. 1, and Materials and Methods). After completing the five-phase operant conditioning paradigm (Fig. 2 and Materials and Methods), mice were trained to respond to brief (0.06 s) stimuli via a three-phase process where flash duration is progressively shortened from 2 to 0.25 s and finally to 0.06 s (Fig. 3, and Material and Methods). Flash intensities were kept constant at a relatively bright level of 400 R*/rod/s for all flash durations. Here, the sensitivity index, *d′* (Eq. 1), was used to measure performance. Mice reliably and reproducibly responded to these short flashes as shown by the low day-to-day variability in *d′* that each mouse exhibited (Fig. 3*A*). On average, mice took 14.2 ± 1.7 d to complete this training process. We required no minimal *d′*. Despite the lack of enforcement, we found that *d′* was similar between mice at each flash duration (Fig. 3*B*). Furthermore, this level of performance was high at 2 s flash (average *d′ = *3.05) and 0.26 s flash (average *d′ = *2.05; Fig. 3*B*). Performance was lower at 0.06 s flash but was still suprathreshold (average *d′ = *1.78), where threshold is defined as *d′ = *1 (Fig. 3*B*). Additionally, the variability in *d′* between mice at 0.06 s was minimal (inter-mouse SD *= *0.32) compared with the 0.26 s condition (inter-mouse SD = 0.71). We also found that the difference in CV between mice at 0.06 s was the smallest out of all the three training conditions (CV SD = 1.3%; Fig. 3*C*). A demonstration of a mouse (Mouse 3) completing the 1AFC for a 0.06 s stimulus is given in Movie 1. From this, we concluded that by the time mice are finished with single-intensity training, they are all capable of completing the 1AFC.

**Movie 1.** A short video of Mouse 3 completing the 1AFC task. The stimulus used is a bright (∼24,000 R*/rod/s) 0.06 s stimulus where *λ ***= **496 nm. The flash was given against a dark background; any light sources other than the stimulus (which appears as a green flash) are infrared. This includes the infrared beam used to detect tray visits and a central infrared light source used to make the mouse visible as it behaves in the dark. The mechanical whirring is the delivery of the reward for correct responses, which is followed by a short high-pitched tone to indicate that the mouse can initiate the next trial. Trials were initiated by the mouse visiting the reward tray. If the mouse correctly visits the right nose-poke, i.e., a hit, it receives a reward; if not, this is a miss, and the mouse does not receive a reward. If the mouse correctly visits the left nose-poke, i.e., a correct rejection (CR), the mouse receives a reward; if not, this is a false alarm (FA), and the mouse does not receive a reward. We can see that Mouse 3 initially visits the right nose-poke on every trial and either moves to the left nose-poke or goes back to the reward tray. We only counted the last nose-poke visit before returning to the tray as a response. Because the stimulus is so bright, this mouse did not make any misses; however, it does make two FAs. The mouse was not dark-adapted before filming. [View online]

### Multi-intensity experiment performance progresses over time

By the end of the 0.06 s single-intensity training, mice became adept at detecting relatively bright, brief, flashes of light of a single intensity. However, mice still demonstrated improvement in performance over time while completing multi-intensity experiments (see Materials and Methods for details of multi-intensity experiments). For example, Mouse 1 initially performed poorly in Condition 1, with most *d′* values <1 (Fig. 4*A*). However, as Mouse 1 was repeatedly tested under Condition 1, its performance gradually improved. Eventually, it performed the task so well that the *d′* values for all intensities fell well above 1. Mouse 1 was then quickly moved along to Conditions 2 and 3, as all *d′* values were well above 1 for both conditions (Fig. 4*B,C*). For the next condition (Day 8, data not shown), Mouse 1 was moved to a dimmer condition, but could not perform consistently. Thus, light levels were raised to Condition 4. Mouse 1 appeared to operate near threshold initially but then improved during the following session (Fig. 4*D*). Under Condition 5, Mouse 1 met our criteria for operating near threshold and stability (Fig. 4*E*). Performance did not continue to improve significantly as demonstrated by two metrics. First, the CV values for *d′* averaged across 3 d all remained well under 45% (which our pilot studies suggested to be the upper CV limit for stabilized psychometric functions; Fig. 4*B*). Second, thresholds taken from 3 d moving averages of *d′* differed at most by 0.2 log units. Therefore, only data collected during the last 3 d of Condition 5 were used in further analysis. Similar behaviors were observed with all mice as we gradually lowered the intensity range of these experiments until our stability and performance criteria were met (see Materials and Methods). Thus, we conclude that we successfully developed a conditioning, training, and testing program to obtain reliable estimates of the absolute visual threshold in mice.

### Absolute visual threshold of mice to full-field flash stimuli

#### Signal detection theoretic thresholds are independent of response bias

**Figure 5.**
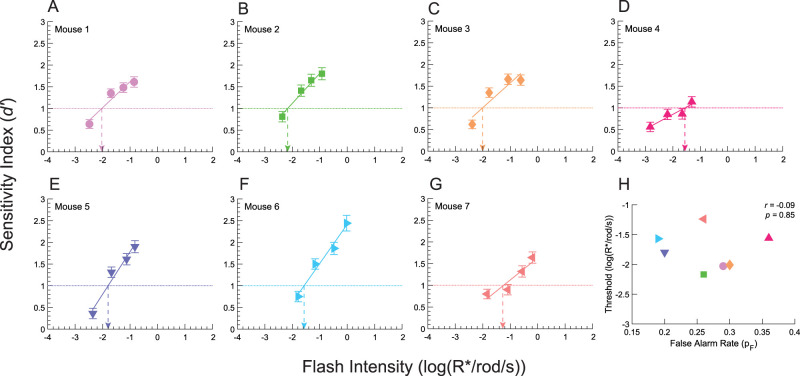
Signal detection theoretic estimation of absolute visual threshold. ***A–G***, *d′-*psychometric functions for each mouse. The solid lines are psychometric functions obtained by fitting *d′* to Equation 2. The dotted lines show *d′ = *1, i.e., the performance level at threshold. Threshold intensities are indicated by arrows. Error bars are *d′* standard errors. ***H***, Threshold intensities plotted against false alarm rates (*p*_F_). Threshold intensities are not correlated with *p*_F_ (Pearson's correlation analysis).

Decision bias is an observer's a priori tendency to respond “yes” or “no,” regardless of stimulus presentation. This can distort percentage-based measures of detectability (Macmillan and Creelman, 2005). In the theory of signal detection, *d′* is a detectability measure that acts independently of bias (Fig. 1*C,D*; Tanner and Birdsall, 1958; Green and Swets, 1966; Macmillan and Creelman, 2005). Thus, to obtain bias-independent estimates of the murine absolute visual threshold, we fit pooled data with a log-linear psychometric function that relates *d′* to flash intensity, from the last three to four multi-intensity experiments that met stability criteria (Eq. 2). Values of *d′* increased monotonically with flash intensity and were well fit by log-linear psychometric functions (*R^2 ^> *0.9 for all fits, Fig. 5*A–G*). From these fits, we obtained estimates of the absolute visual threshold (*I*_T_) for each mouse, where threshold is the intensity where *d′ *= 1 (Table 1). The average of the threshold estimates was −1.77 log(R*/rod/s) [SD *= *0.33 log(R*/rod/s)] or 1.7 × 10^−2^ R*/rod/s. Given a flash duration of 0.06 s, this is equivalent to 1.02 × 10^−3^ R*/rod or 1 photoisomerization in every 980 rods in each retina for our full-field, binocular stimulus. The slope of the *d′*-psychometric functions (*β*) indicates the sensitivity of mice to systematic increases in flash intensity (Macmillan and Creelman, 2005). We found that the average value of the slope, *β*, was 0.66 *d′*·log(R*/rod/s)^−1^ (Table 1). These values ranged from 0.34 to 0.98 (SD *= *0.22). We found no relationship between *β* and threshold (Pearson's *r = *0.03, *p *= 0.96).

**Table 1.** *d*′-psychometric function parameters and false alarm rates

Animal ID	Threshold intensity^a^ log_10_(R*/rod/s)	Psychometric function slope^a^ (*d′*·log_10_(R*/rod/s)^−1^)	False alarm rate (*p*_F_)^b^
Mouse 1	−2.03 ± 0.13 (−2.26, −1.74)	0.60 ± 0.08 (0.47, 0.78)	0.29 ± 0.016 (0.26, 0.32)
Mouse 2	−2.17 ± 0.15 (−2.42, −1.88)	0.69 ± 0.12 (0.49, 0.95)	0.26 ± 0.018 (0.23, 0.30)
Mouse 3	−2.01 ± 0.14 (−2.25, −1.72)	0.57 ± 0.08 (0.44, 0.75)	0.30 ± 0.016 (0.27, 0.33)
Mouse 4	−1.56 ± 0.32 (−2.03, −0.72)	0.34 ± 0.09 (0.17, 0.54)	0.36 ± 0.020 (0.32, 0.39)
Mouse 5	−1.80 ± 0.09 (−1.95, −1.60)	0.97 ± 0.10 (0.81, 1.20)	0.20 ± 0.016 (0.17, 0.24)
Mouse 6	−1.57 ± 0.11 (−1.78, −1.37)	0.90 ± 0.11 (0.71, 1.13)	0.19 ± 0.016 (0.16, 0.22)
Mouse 7	−1.24 ± 0.17 (−1.65, −0.96)	0.51 ± 0.09 (0.32, 0.67)	0.26 ± 0.018 (0.22, 0.29)
Average	−1.77 ± 0.23 (−2.07, −1.47)	0.66 ± 0.19 (0.45, 0.86)	0.27 ± 0.097 (0.21, 0.32)

Parameter estimates of the *d′*-psychometric function obtained via nonlinear least squares (i.e., *I*_T_ and *β*) ± standard errors for each mouse. Additionally, we show the directly measured false alarm rates obtained from pooling across experiments.

a Standard errors and 95% CI for each mouse, were obtained via nonparametric bootstrapping.

b Standard errors are binomial standard errors and were used to calculate the normal approximation of the 95% CI.

An observer's bias is reflected in their false alarm rate (*p*_F_; Gescheider, 1997; Macmillan and Creelman, 2005). Our mice had an average *p*_F_ of 0.27, ranging from 0.2 to 0.36 (SD = 0.06; Table 1). Our threshold estimates should be independent of these false alarm rates, because *d′* is a bias-independent measure of visual sensitivity (Tanner and Swets, 1954; Swets et al., 1961). To test for an association between *p*_F_ and threshold, we could have either performed linear regression or correlation analysis. Given that both *p*_F_ and log-threshold are dependent variables, regression was not applicable. Thus, we performed a correlation analysis on log-threshold and *p*_F_. We found no significant relationship between log-threshold and *p*_F_ (Pearson's *r = *−0.11, *p = *0.81; Fig. 5*H*). However, we did find that *β* values were strongly correlated with *p*_F_ (Pearson's *r = *−0.92, *p = *0.003). Thus, we conclude that our *d′*-obtained absolute visual thresholds were independent of response bias but that *d′-*psychometric function slopes were strongly dependent on response bias.

#### High-threshold theoretic thresholds are dependent on bias

Because *d′* is not typically used when estimating absolute visual threshold, we compared our approach to the more conventional high-threshold theory approach. Unlike the theory of signal detection, high-threshold theory considers *p*_H_ and *p*_F_ to be independent; *p*_F_ simply reflects a constant *guess rate* that leads to performance bias (Blackwell, 1963; Green and Swets, 1966; Macmillan and Creelman, 2005). Consequently, according to high-threshold theory, one can obtain a bias-free measure of sensitivity by correcting the observed hit rate for guessing (Green and Swets, 1966; Gescheider, 1997; Macmillan and Creelman, 2005).

**Figure 6.**
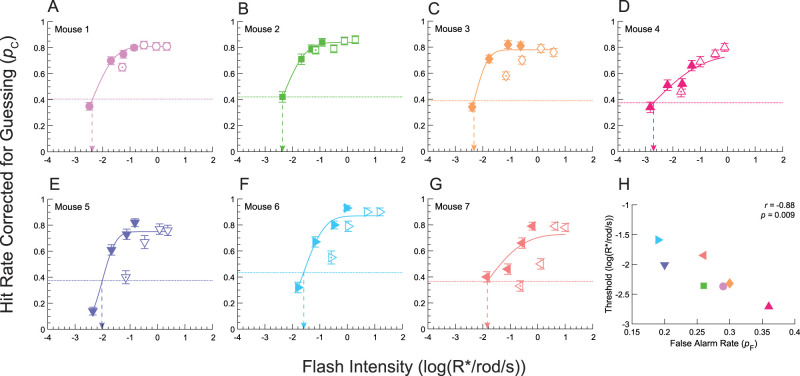
High-threshold theoretic estimation of absolute visual threshold. ***A–G***, *p*_C_*-*psychometric functions for each mouse. Hit rates corrected for guessing (*p*_C_) plotted as a function of log(R*/rod/s). The solid symbols are from the low-intensity condition, and the empty symbols are taken from the high-intensity condition, i.e., asymptotic performance levels. The solid lines are psychometric functions obtained by fitting Equation 4 to solid symbols and Equation 5 to empty symbols. The dotted lines show threshold performance as given by *μ* in Equation 4. Threshold intensities are indicated by arrows. Error bars are standard errors. ***H***, Threshold intensities plotted against false alarm rates (*p*_F_). Threshold intensities are correlated with *p*_F_ (Pearson's correlation analysis).

Figure 6*A–H* shows the *p*_H_ corrected for guessing (*p*_C_) for each mouse as a function of log intensity. Here, the *p*_H_ was corrected for guessing via Equation 3 (Pelli, 1985) using the false alarm rates listed in Table 1. Solid-colored symbols are from low-intensity conditions, and empty symbols are from high-intensity conditions (asymptotic performance level or APL; see Materials and Methods for details). Empty symbols with dots correspond to the performance at the lowest APL intensity and were not used for fitting because they underestimated the corresponding values obtained with low-intensity conditions. This underestimation was most likely due to desensitization by the brighter flashes in APL. For each mouse, the *p*_C_ rose monotonically over the first 1–1.5 log units until it reached an asymptotic level that is independent of stimulus intensity and sits well below perfect detection (*p*_C _= 1). High-threshold theory postulates that asymptotic behavior at APL arises due to observer lapses.

To compare our signal detection theoretic approach to the classical high-threshold theoretic approach, we estimated the thresholds of *p*_C_ by modeling *p*_C_as a cumulative Gaussian function with a mean, *μ*, that corresponds to threshold, and a standard deviation, *σ*, that is inversely proportional to the slope; additional terms, *p*_L_ and *p*_F_, are included to account for observer lapses, *p*_L,_ and guesses, *p*_F_(Eq. 4; Kingdom and Prins, 2010). Since we have already corrected for guessing, we set *p*_F_ = 0. To estimate the three remaining parameters, we used joint asymptotic performance lapse estimation, which minimizes statistical bias in our parameter estimates (Prins, 2012). This method involves simultaneously fitting low-intensity *p*_C_ values to Equation 4 and APL *p*_C_ values to Equation 5 (see Materials and Methods; Prins, 2012). The resulting psychometric functions (Fig. 6) gave threshold estimates that were systematically lower than those obtained via the signal detection theoretic approach (Table 2). The average threshold (*μ*) is −2.17 log(R*/rod/s) (SD *= *0.37) or 6.6 × 10^−3^ R*/rod/s. Given a flash duration of 0.06 s, this corresponds to 4.0 × 10^−4^ R*/rod or one photoisomerization per 2,517 rods. The *σ* parameter reflects the “noisiness” of the sensory transduction process (Kingdom and Prins, 2010). The average *σ*, which is inversely related to the slope, was 0.73 log(R*/rod/s) (SD = 0.25). Lapse rates (*p*_L_) were, on average, 0.21 (SD = 0.06). Although a *p*_L_ of 0.2 is high for human observers (Wichmann and Hill, 2001; Prins, 2012), our *p*_L_ values are comparable, if a little high, to those in prior rodent psychophysical studies (Hayes and Balkema, 1993a, b; Naarendorp et al., 2010; Pisupati et al., 2021). Further, our calculated *p*_L_ may be inflated due to our correction for guessing, which will depress *p*_H_ values.

**Table 2.** Corrected for guessing psychometric function parameters

Animal ID	Mean parameter (*μ*)^a^ log_10_(R*/rod/s)	Standard deviation parameter (*σ*)^a^ (*d′*·log_10_(R*/rod/s)^−1^)^−1^	Lapse rate^b^ (*p*_L_)
Mouse 1	−2.37 ± 0.10 (−2.54, −2.15)	0.65 ± 0.17 (0.08, 0.93)	0.19 ± 0.018 (0.15, 0.22)
Mouse 2	−2.36 ± 0.12 (−2.90, −2.22)	0.69 ± 0.16 (0.43, 1.10)	0.16 ± 0.020 (0.13, 0.21)
Mouse 3	−2.32 ± 0.06 (−2.49, −2.21)	0.40 ± 0.13 (0.06, 0.61)	0.22 ± 0.015 (0.20, 0.26)
Mouse 4	−2.71 ± 0.31 (−3.52, −2.32)	1.42 ± 0.57 (0.82, 3.35)	0.25 ± 0.027 (0.21, 0.31)
Mouse 5	−2.01 ± 0.08 (−2.17, −1.86)	0.39 ± 0.08 (0.26, 0.56)	0.25 ± 0.020 (0.20, 0.29)
Mouse 6	−1.59 ± 0.08 (−1.72, −1.41)	0.61 ± 0.12 (0.41, 0.85)	0.13 ± 0.017 (0.10, 0.16)
Mouse 7	−1.89 ± 0.24 (−2.72, −1.59)	1.01 ± 0.20 (0.75, 1.62)	0.29 ± 0.021 (0.26, 0.34)
Average	−2.18 ± 0.25 (−2.55, −1.83)	0.73 ± 0.25 (0.40, 1.07)	0.21 ± 0.057 (0.16, 0.27)

Parameter estimates of the high-threshold psychometric function obtained via nonlinear least squares (i.e., *μ*, *σ*, and *p*_L_) ± standard errors for each mouse. For each individual parameter estimate, standard errors and 95% CI were obtained via nonparametric bootstrapping *d′*.

a Standard errors and 95% CI for each mouse, were obtained via nonparametric bootstrapping.

b Standard errors are binomial standard errors and were used to calculate the normal approximation of the 95% CI.

To determine whether high-threshold theoretic threshold estimates are sensitive to response bias, we ran a correlation analysis of log(*μ*) and corresponding *p*_F_ values. We found a significant, inverse, relationship between false alarm rate and log-threshold (Pearson's *r = *−0.88, *p = *0.009; Fig. 6*H*). Further, *σ* values were not correlated with the false alarm rate (Pearson's *r = *0.62, *p = *0.14). Thus, we conclude that high-threshold theoretic threshold estimates are sensitive to response bias, even when accounting for guessing.

#### The theory of signal detection explains inter- and intrasession variation in detection behavior

**Figure 7.**
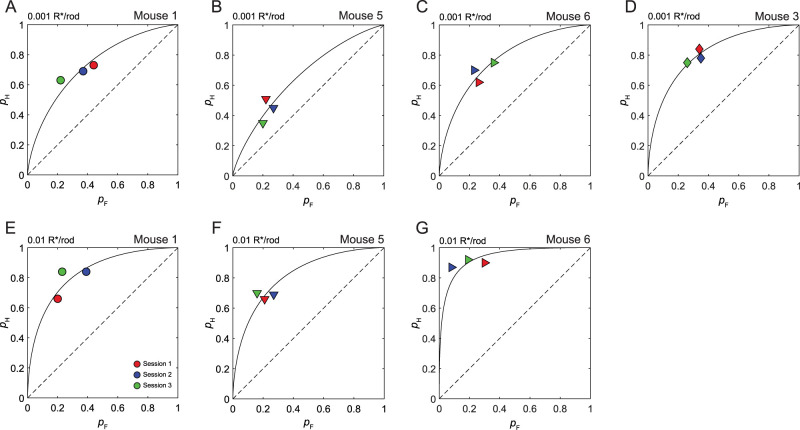
The theory of signal detection accounts for intersession variability in detection. Hit rate (*p*_H_) and false alarm rate (*p*_F_) pairs plotted in ROC space from three consecutive experimental sessions under low-intensity (***A–C***) and high-intensity (***D–G***) conditions. Each session was conducted on a different day. For each mouse and condition, the session number corresponds to a different symbol color; the color code is common to all mice and conditions. Also plotted are isosensitivity curves implied by the *d′* that corresponds to the data pooled from all three sessions. The corresponding *d′* values for each plot were as follows: ***A***, *d′ *= 0.89; ***B***, *d′ *= 0.58; ***C***, *d′ *= 1.07; ***D***, *d′ *= 1.28; ***E***, *d′ = *1.38; ***F***, *d′ *= 1.28; ***G***, *d′ *= 2.16. Note that only three mice (Mice 1, 5, and 6) completed the high-intensity condition, as we could not obtain reliable data for Mouse 3 under the high-intensity condition.

To further investigate decision bias on an intersession basis, four mice completed additional single-intensity experiments. Here, mice completed the 1AFC detection task under a “low-intensity” condition at threshold (∼0.001 R*/rod) or a “high-intensity” condition, near threshold (∼0.01 R*/rod). From three mice, we obtained hit and false alarm rates for three consecutive experimental sessions at both threshold and near-threshold intensities. For one mouse (Mouse 3), we were only able to obtain reliable data for the threshold intensity condition. From these experimental sessions, we generated receiver operating characteristic (ROC) plots, which plots *p*_H_ as a function of *p*_F_ (Fig. 7). The theory of signal detection predicts that *p*_H_ and *p*_F_ pairs for a given stimulus should fall along an isosensitivity curve, where all the points that fall along the curve share a common *d′*. While the *d′* remains constant, it is the observer's decision bias that changes along the isosensitivity curve. According to the theory of signal detection, these changes in bias reflect the observer changing their criterion location (Fig. 1*D*; Green and Swets, 1966; Macmillan and Creelman, 2005).

In Figure 7, we plotted the *p*_H_ and *p*_F_ pairs from each session along with the isosensitivity curve that corresponds to the pooled *d′* from all three sessions. For illustrative purposes, we consider Mouse 1 (Fig. 7*A,D*) in detail. Looking first at the high-intensity condition (Fig. 7*A*), Mouse 1 displayed variation in *p*_H_ and *p*_F_on a session-to-session basis. However, the *p*_H_ and *p*_F_ covaried in such a way that they moved along a single isosensitivity curve, corresponding to a *d′ *= 1.39 (Fig. 7*A*). Looking at the low-intensity condition (Fig. 7*D*), session *p*_H_ and *p*_F_ covaried such that they also fall along an isosensitivity curve, corresponding to a *d′ *= 0.89. In contrast, Mouse 5 (Fig. 7*B,E*) did not display as much session-to-session variation in *p*_F_ as Mouse 1. However, in both the high- and low-intensity conditions, the *p*_H_ and *p*_F_ pairs still clustered about the isosensitivity curve (Fig. 7*B,E*). Overall, *p*_H_ and *p*_F_ pairs either fell along or clustered about their corresponding isosensitivity curves in all mice (Fig. 7). Thus, we can qualitatively infer from the ROC plots that the theory of signal detection explains a good deal of variation in session-to-session performance.

**Figure 8.**
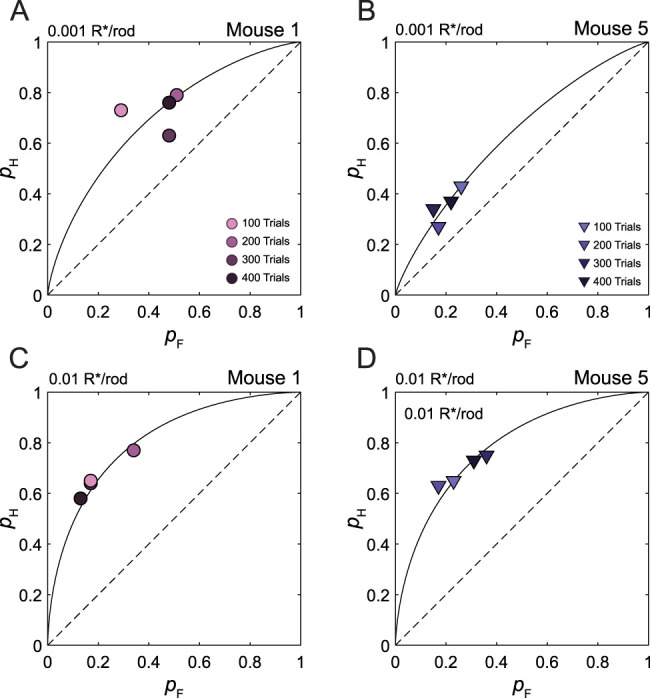
The theory of signal detection accounts for intrasession variability in detection. Representative ROC plots of hit rate (*p*_H_) and false alarm rate (*p*_F_) pairs taken from single experimental sessions of Mice 1 (***A*** and ***C***) and 5 (***B*** and ***D***). ***A*** and ***B*** show plots of data obtained under the low-intensity condition, whereas ***C*** and ***D*** show plots of data obtained under the high-intensity condition. In each plot, each point reflects data taken from trials 0 to 100, 100 to 200, 200 to 300, and 300 to 400. Symbols are color-coded such that the tint becomes darker as the number of trials increases. Also plotted are isosensitivity curves implied by the *d′* that corresponds to the pooled data of that session. The corresponding *d′* values were as follows: ***A***, *d′ *= 0.76; ***B***, *d′ *= 0.47; ***C***, *d′ = *1.24; ***D***, *d′ *= 1.12. Note that each experimental session was conducted on a different day.

To investigate whether the theory of signal detection also explained *intra*session variation in detection behavior, we binned *p*_H_ and *p*_F_ into 100 trial blocks and plotted the resultant *p*_H_ and *p*_F_ pairs in an ROC plot. In Figure 8, we show representative ROC curves from Mouse 1 (Fig. 8*A,C*) and Mouse 5 (Fig. 8*B,D*). Each plot comes from a single session under either the high- or low-intensity condition. In both mice, there was variation in both *p*_H_ and *p*_F_ within a single experimental session. However, as the *p*_H_ and *p*_F_ changed over the course of a session, they covaried along the isosensitivity curve that corresponded to the *d′* for that session (Fig. 8). In particular, we can see that the *p*_H_ and *p*_F_ pairs in Figure 8, *A*, *B*, and *D*, very closely followed their corresponding isosensitivity curves. In Figure 8*C*, we can see that Mouse 1's low-intensity condition data did not follow the isosensitivity curve as closely. However, the data still clustered about the corresponding isosensitivity curve. The power of this analysis comes from the fact that the implied ROC curve is determined entirely by the *d′*, no free parameters were used to plot the ROC curve. Thus, we can qualitatively infer that the theory of signal detection explains most of the variation within experimental sessions as well. In all, these findings work to further validate our signal detection theoretic approach.

## Discussion

In this study, we investigated the effect of response bias near absolute visual threshold, using a 1AFC task. We found that the theory of signal detection provides estimates of absolute visual threshold that are independent of response bias. Conversely, the more conventional, high-threshold theoretic, approach is not independent of response bias. Furthermore, using ROC plots, we have found that the theory of signal detection accounts for inter- and intrasession variability in detection behavior. These results have important implications for our understanding of vision near the absolute threshold, and, more broadly, the influence of bias in perceptual judgments.

### The theory of signal detection provides a bias-independent estimate of murine absolute visual threshold

We have shown that our signal detection theoretic approach to the estimation of the murine absolute visual threshold is independent of false alarm rate. In contrast, the more conventional high-threshold theoretic approach results in estimations of visual threshold that are inversely related to false alarm rates. The high-threshold theory has been repeatedly shown to be an inadequate theory of vision, yet it continues to be widely used in human and animal psychophysical experiments (Swets, 1961; Laming, 2013). Here, we demonstrate the superiority of the theory of signal detection in mice. We also reinforce the importance of using the forced choice procedure in murine perceptual experiments, as the forced choice procedure is the only psychophysical method that allows for direct measurement of the false alarm rate (Macmillan and Creelman, 2005; Kingdom and Prins, 2010; Umino et al., 2018).

Several different methods have been used to estimate the murine absolute visual threshold. An early method is the six-arm water maze, wherein mice must identify which of the six maze arms contains an exit ramp set against a black background (Hayes and Balkema, 1993a, b; Sampath et al., 2005; Nathan et al., 2006). In these experiments, mice were tasked with detecting a dark target in a white, diffusely lit maze. Therefore, relative *decrement* thresholds are being measured, not absolute increment thresholds.

Smeds et al. have recently developed an improved version of this method, wherein the arm with the exit ramp is illuminated with a continuously illuminated spot stimulus (Smeds et al., 2019). Using sophisticated eye and body tracking technology, Smeds et al. model how ganglion cell activity is used in visual search tasks (Smeds et al., 2019). Using this method, Smeds et al. estimated the murine absolute threshold to be ∼0.05 R*/rod/s, which is approximately three times higher than the threshold intensity reported here for a flash stimulus (Smeds et al., 2019). However, direct comparison with our results is complicated by the stimulus's continuous illumination and by the fact that in this paradigm the size of the stimulus changes as the mouse moves about the maze. This means that illumination levels are changing with the square of the distance to the stimulus. Furthermore, with this approach, stimulus intensities are not randomized; instead, light stimuli are progressively dimmed until the mouse performs at chance (Smeds et al., 2019). This sequential dimming of the light stimulus, also known as the method of limits, can lead to inaccurate estimates of threshold due to habituation (Gescheider, 1997).

Stimulus randomization was first used in an early approach, where food-restricted mice were conditioned to pull a lever when a light stimulus was given in return for a fruit juice reward (Herreros De Tejada et al., 1997). With this method, Herreros De Tejada et al. estimated the threshold to be −5.5 log cd m^−2^, which is, according to the calculations of Naarendorp et al., ∼2.3 × 10^−3^ R*/rod/s (Herreros De Tejada et al., 1997; Naarendorp et al., 2010). As a stimulus, Herreros De Tejada et al. used a 5 s flashing stimulus with a two-third duty cycle (Herreros De Tejada et al., 1997), which is equivalent to ∼3.5 s worth of light stimulus per trial. Both Smeds et al. (2019) and Herreros De Tejada et al. (1997) utilized prolonged stimuli in time, which requires complex analysis to determine how much light was required for the mouse to make a decision (Herreros De Tejada et al., 1997; Smeds et al., 2019).

The current gold standard for measuring the absolute visual threshold of mice is Naarendorp et al.'s operant behavioral assay (Naarendorp et al., 2010). Naarendorp et al. took advantage of a naturally occurring behavior of the lab mouse, i.e., nocturnal wheel-running. Water-restricted mice would be presented with a single flash of light above their wheel, which they were trained to associate with the availability of water. By tallying when mice would visit their water spout, Naarendorp et al. obtained frequency-of-seeing curves (Naarendorp et al., 2010). Further, by restricting the location of the mouse to the running wheel, Naarendorp et al. precisely localized the flash stimulus. Using this method, the estimated absolute threshold is ∼6 × 10^−3^ R*/rod for a stimulus covering 2,000 rods (Naarendorp et al., 2010). If we assume perfect temporal summation, our average threshold intensity, in terms of R*/rod, is approximately sixfold lower.

Our flashes were 0.06 s in duration and covered the entire retina, whereas those of Naarendorp et al. are ≤1 × 10^−3^ s in duration and spatially localized to 800–3,000 rods (Naarendorp et al., 2010). Thus, the difference between our average threshold estimate and that of Naarendorp et al. is most likely due to differences in the spatiotemporal dimensions of our stimuli. Both flash stimuli fall within the integration time of rods (∼0.25 s; Chen et al., 2000; Burns et al., 2002; Krispel et al., 2006) and within the psychophysical critical duration of humans (∼0.1 s; Barlow, 1958; Zuidema et al., 1981; Sharpe et al., 1988). This suggests that differences in the duration of the stimulus may not contribute significantly to the difference in R*/rod.

Thus, the difference in threshold is most likely due to differences in the spatial features of the stimulus. Recently, Dey et al. found that full-field stimulation of the human retina lowers the absolute visual threshold by ∼2,930× compared with the classic measurements of Hecht, Shlaer, and Pirenne, whose stimulus subtended ∼500 rods (Hecht et al., 1942; Dey et al., 2021). Our findings are consistent with Dey et al.'s results insofar as the murine absolute threshold is lower for a full-field stimulus. However, the degree to which threshold is lowered is much larger in the human retina than in the murine retina. It is noteworthy that mice display supralinear spatial summation at absolute visual threshold, a phenomenon not seen in human psychophysics (Barlow, 1958; Zuidema et al., 1981; Sharpe et al., 1993; Naarendorp et al., 2010), suggesting that spatial summation may work differently in murine and human retinae. This would be consistent with the disparity in the make-up of ganglion cell types between murine and human retinae and/or postretinal processing (Hahn et al., 2023).

Our visual stimuli were binocular and full field. This differs from most studies of the human absolute visual threshold, which have been measured monocularly in the sensitive, rod-dominant, peripheral retina using spatially localized stimuli (Hecht et al., 1942; Barlow, 1956; Sakitt, 1972; Teich et al., 1982; Sharpe et al., 1988). Our use of a binocular stimulus is in line with previous studies of the murine absolute threshold (Herreros De Tejada et al., 1997; Naarendorp et al., 2010; Smeds et al., 2019), which could result in binocular summation. In humans, binocular summation scales thresholds by a factor of two or less and can be considered in the context of probability summation (when binocular stimuli are not simultaneous or applied in noncorresponding areas) or in terms of quadratic summation and linear physiological summation for corresponding stimuli (Pirenne, 1943; Thorn and Boynton, 1974; Legge, 1984). To our knowledge, binocular summation has not been studied in mice, where >95% of retinal projections are contralateral and <5% are ipsilateral, which leads to <20% binocular vision localized to the temporal retina (Johnson et al., 2021). This suggests that to varying degrees, both probability and physiological summation mechanisms might shape the murine threshold. Thus, the maximum that binocular summation could lower our threshold estimates is by 0.3 log units, well within the variability of our measurements, and should therefore have a negligible effect on our estimates of threshold.

We compare our threshold with the human threshold estimated by Dey et al. (2021) whose stimulus parameters most closely match our own, insofar as they used a full-field stimulus. Dey et al. estimated the human absolute visual threshold for a brief, monocular, full-field flash to be ∼1,000 R*/retina, distributed over ∼92 million rods (Dey et al., 2021). Given our threshold estimate of 1.02 × 10^−3^ R*/rod, and that the mouse retina has 6.4 million rods (Jeon et al., 1998), the mouse threshold is ∼6,500 R*/retina in total. It is compelling that murine and human full-field absolute thresholds differ by only 6.5-fold in terms of total R*/retina, while if we express this in terms of R*/rod, the human threshold is two orders of magnitude lower than that of mice (Dey et al., 2021). This suggests that, despite differences in anatomy, a similar number of R* are required across species for absolute visual threshold for full-field stimuli.

### The one-alternative forced choice assay provides direct measurement of false alarms

During threshold experiments, false alarm rates ranged from 0.19 to 0.36. The false alarm rate is typically treated as a “nuisance parameter,” which distorts behavioral data. Thus, little attention has been paid to the false alarm rate in prior studies of mouse absolute visual threshold. Of the studies mentioned, Naarendorp et al. (2010) is the only one to report false alarm rates (Naarendorp et al., 2010). While Naarendorp et al. did not directly measure false alarm rates in the same way we did, they used Sakitt's threshold model to estimate false alarm rates (Sakitt, 1971; Naarendorp et al., 2010), which range from ∼0.01 to 0.03. These estimates were considerably lower than our false alarm rates. This discrepancy may originate from the nature of the stimulus used (full-field vs localized) or the nature of the task, such that mice performing the running-wheel task adopt a stricter criterion.

Multialternative forced choice assays, like the six-arm water maze, are theoretically bias-free (Kingdom and Prins, 2010; Carandini and Churchland, 2013; Smeds et al., 2019; although not necessarily bias-free in practice; Klein, 2001). However, such assays preclude the ability to measure the false alarm rate, which holds important information concerning how signal and noise are processed by the observer (Barlow, 1956; Green and Swets, 1966; Sakitt, 1972, 1974; Makous, 1990; Donner, 1992; Koenig and Hofer, 2011). The one-alternative forced choice assay used in this study gives us the best of both worlds: We can directly assess bias while also mitigating its effects via the use of the theory of signal detection (Green and Swets, 1966; Umino et al., 2018). We put this into practice by generating ROC curves.

### ROC plots validate the signal detection theoretic approach

We have demonstrated that the theory of signal detection accounts for inter- and intraexperimental session variability. Specifically, we found that pairs of *p*_H_ and *p*_F_, either taken from the entire session or within a session, followed the isosensitivity curves implied by the corresponding *d*′ value. These results serve to validate our signal detection theoretic approach to measuring the murine absolute visual threshold.

It is important to note that a key assumption for using the *d′* is that the underlying noise is normally distributed. One can infer this to be the case if *p*_H_ and *p*_F_ pairs follow the isosensitivity curve predicted by the theory of signal detection (Green and Swets, 1966). This assumption is at odds with the widely accepted Poisson model of threshold vision, which models both noise and signal as Poisson distributed (Hecht et al., 1942; Barlow, 1956; Makous, 1990; Donner, 1992; Field et al., 2005; Koenig and Hofer, 2011). The Poisson model of threshold vision is based on the fact that the variability of both the light stimulus (i.e., signal) and the spontaneous isomerization of rhodopsin (i.e., noise) is Poisson limited (Hecht et al., 1942; Barlow, 1956; Sakitt, 1972; Field et al., 2005). The critical assumption is that this Poisson process is the *only* source of noise with which the observer contends at absolute threshold (Barlow, 1956; Field et al., 2005).

That our data fell along, or clustered about, the isosensitivity curve shows that our use of the *d′* is a valid measure of visual sensitivity near absolute threshold. Further, because our isosensitivity curves assumed normally distributed signal and noise, our results raise the possibility that the nature of the noise limiting detection may be normally, not Poisson, distributed. One possible explanation for this is that when the stimulus used is a full-field flash stimulus, many receptive fields will be activated at once. As such, the mouse must monitor many independent sources of noise. Via the central limit theorem, this noise, and thus any signal added to it, will be approximated by a normal distribution (Green and Swets, 1966). However, this effect may not be unique to our stimulus. The rating data obtained from Sakitt's experiments (where small stimuli were used) are fit by models assuming normally distributed noise and signal better than assuming Poisson-distributed noise and signal (Sakitt, 1974; Makous, 1990). Although the Poisson statistics seen at the level of the photoreceptor are maintained at the level of the ganglion cell (Barlow et al., 1971; Chichilnisky and Rieke, 2005; Ala-Laurila and Rieke, 2014), additional, additive, neural noise of central origin may play a significant role in limiting the absolute visual threshold (Barlow, 1977). However, Poisson-distributed signal and noise can also be used to generate isosensitivity curves (Thibos et al., 1979; Makous, 1990). Although our data fall along the isosensitivity curve, it does not extend far enough to rule out the possibility of normal or Poisson-distributed signal and noise.

## Conclusions

We have demonstrated the applicability of the theory of signal detection to the problem of the murine absolute visual threshold. By properly measuring the false alarm rate, we have obtained threshold intensity estimates that are independent of response bias. Further, we have shown that the theory of signal detection provides an adequate explanation of variation observed in murine detection performance. Our findings are important because they demonstrate that response bias plays a significant role in mouse detection tasks at absolute visual threshold and that the distorting effects of bias can be eliminated by using signal detection theoretic measures, such as the *d′*.

## Synthesis

Reviewing Editor: Matthew Grubb, King's College London Decisions are customarily a result of the Reviewing Editor and the peer reviewers coming together and discussing their recommendations until a consensus is reached. When revisions are invited, a fact-based synthesis statement explaining their decision and outlining what is needed to prepare a revision will be listed below. The following reviewer(s) agreed to reveal their identity: NONE.

Both Reviewers are positive about the manuscript overall. They also suggest several issues that need to be addressed. Please ensure that the revised submission covers all of the Reviewers' points. In particular, on consultation we clarified that Reviewer 1's request (under 'Discussion') to 'generate models' involves conducting new analyses by combining data from the current study with published data and fitting models to the integrated dataset.

Reviewer 1:

This study evaluates the effect of response bias on the absolute visual threshold and shows that the signal detection better eliminates the effect than the conventional high threshold approach. The findings are interesting. In the following, I outline my key comments and questions:

Introduction: It would be worth discussing that mouse rods and human rods share similar characteristics and so mouse studies can inform human studies on the absolute visual threshold.

Cite the human studies that show variable thresholds.

Most of the information in the last paragraph of Introduction can be moved to Methods. At the end of the paragraph, please outline the key findings before stating what the findings suggest.

Methods:

Line86: State the [Authors' University]

L97: Please expand on the LED characteristics. What was the full width at maximum output? How were the spectral properties and intensity levels calibrated (using what instrument)?

L118: Please clarify for the reader, why such a brief stimulus was used (60 ms)? How does the choice of the stimulus duration affect the findings and comparisons with other studies? This is worth discussing in Discussion if you have not already.

Throughout the manuscript (including Figures), it would be informative to provide the flash intensity in photons/μm2/sec (OR photons/m2/s) as well. The figures could report these values across the top x-axis.

Were all mice the same age? If not, they may have different lens densities and so retinal irradiances. Was this considered in the analysis?

I guess the experiments were conducted under binocular viewing (both eyes open). This means the number of photons present in the light can be assumed to be divided in to two eyes because the same photon cannot enter both eyes. How was this accounted for in the computations? By per steradian visual angle calibration, pupil size? The authors also need to consider whether this is automatically accounted for by the visual system with binocular summation. If so, does this affect the estimates of photon absorbed in XX number of rods for absolute threshold?

Results:

Do the first two sections fit better in Methods?

L375: How did the authors come to 1 photoisomerization in every 980 rods? The reader would appreciate the assumptions and calculations involved in this interesting outcome.

Given such low number of data points, the ROC fit is not so promising. Could there be an alternative analysis?

Discussion:

The authors assume that false alarm rates in visual psychophysics may arise from variations in stimulus size. This is an interesting thought. Can the authors generate models by combining their data and previous studies to inform the effect of stimulus size and duration on FA rate? This will be a useful addition to psychophysics.

Naarendorp et al show that the absolute threshold is similar between mice and humans when measured in the peripheral retina. Was this the case in the current study too? I think the discussion section too method focussed and needs to be expanded on what new we learn with regards to mouse vision and implications in human vision.

Minor comments:

There are several repetitions in the manuscript. For example, 'The signal detection theoretic approach provides estimates of absolute visual threshold' has been stated at least three times. Please minimise such repetitions to make the manuscript more readable.

Reviewer 2:

In this manuscript the authors are using an elegant 1AFC assay to measure visually guided behavior near detection threshold. Using the theory of signal detection the authors try to understand the impact of response bias near absolute visual threshold. Experiments are elegantly done, and these results will help us understand how decision bias can be a factor in murine psychophysical experiments. It will also have implications for understanding vision near absolute threshold and the major noise components underlying visual detection threshold. There are a few things that could be clarified.

The light stimulus was placed overhead (line 97), indicating stimulus of the ventral retina only. The are significant differences between the dorsal and ventral retina, so this should be clarified. Same goes for the comment on line 535, the light does not seem to cover the whole retina. This could also be taken into account when comparing to previous experiments (Naarendorp 2010, Smeds 2019), where the stimulus was projected to the whole retina.

The mice were handled under dim red light (line 113) which will affect dark adaptation state. How bright was this light and how could it possibly affect the experiment (pigment bleaching)?

For some experiments the conclusions rely on N equal to or less than 3, and bootstrapping is used to get standard errors. The authors should comment on this in more detail, and justify the statistical power of these experiments.

The non-bias data is elegantly extracted and it seems to be normally distributed, as opposed to Poison. Maybe the authors could elaborate somewhat on the implications of this results, are there more additive noise sources controlling threshold the the noise from photoreceptors, is the discrete noise not controlling absolute visual threshold?

Minor comment: the F in pF is not always subscripted.

With a few minor clarifications this beautiful work is more than suitable to be published in eNeuro.

## Author Response

Synthesis of Reviews:

Both Reviewers are positive about the manuscript overall. They also suggest several issues that need to be addressed. Please ensure that the revised submission covers all of the Reviewers' points. In particular, on consultation we clarified that Reviewer 1's request to 'generate models' (under 'Discussion') involves conducting new analyses by combining data from the current study with published data and fitting models to the integrated dataset.

We thank the reviewers for their positive comments. Below we address their concerns. Changes in the manuscript are shown in bold font at the indicated lines.

Reviewer 1:

This study evaluates the effect of response bias on the absolute visual threshold and shows that the signal detection better eliminates the effect than the conventional high threshold approach. The findings are interesting. In the following, I outline my key comments and questions:

1.

Introduction: It would be worth discussing that mouse rods and human rods share similar characteristics and so mouse studies can inform human studies on the absolute visual threshold.

We agree, and we have extended the second paragraph in the introduction to address these shared features in mouse and primate retinas Line 46.

2.

Cite the human studies that show variable thresholds We cite differences in threshold estimates between studies dating from the classic work by Hecht et al in 1942 to more recent work by Tinsley et al in 2016. See lines 33-36.

3.

Most of the information in the last paragraph of Introduction can be moved to Methods. At the end of the paragraph, please outline the key findings before stating what the findings suggest.

We edited the last paragraph of the introduction accordingly. We removed references to the methodology, but kept in some language that justifies the approach - lines 61-67 - and outlined the key finding before stating what it suggests - lines 67 - 70.

4.

Methods:

Line86: State the [Authors' University] 5.

L97: Please expand on the LED characteristics. What was the full width at maximum output? How were the spectral properties and intensity levels calibrated (using what instrument)? The characteristics of the LEDs were measured with a spectral radiometer (Photo Research®, SpectraColorimeter, Model PR-650). The LEDs had a central emission at 496 nm and half-maximal bandwidth of 28 nm. The requested information is in the text on line 97 - 100.

Intensity levels were calibrated using the Graseby S370 optomoter in radiometric mode and positioning the photodiode in the location of the mouse cornea directed at the back of the chamber, the nose-poke-side of the chamber, and ceiling of the chamber. These values were averaged together to arrive at the typical irradiance within the chamber. Radiometric measurements were converted to Q, photon flux density at each cornea (in photons/μm2/s), and then to the average number of photoisomerizations (R*) absorbed per rod per second in each retina using the expression derived by Lyubarsky et al. Full details are given in the Methods lines 295-311 where we describe how we determined retinal irradiance.

6.

L118: Please clarify for the reader, why such a brief stimulus was used (60 ms)? We chose this short duration because it falls within the integration time of the mouse rod and the human psychophysical critical duration. We have made the justification explicit and provide references in lines 123 - 125.

7.

How does the choice of the stimulus duration affect the findings and comparisons with other studies? This is worth discussing in Discussion if you have not already.

This is a very good question; in fact, we have completed a separate, systematic study investigating the effect of duration on absolute threshold. These results have been presented as an abstract at ARVO 2024, and the manuscript is currently in preparation.

In the discussion we have discussed how using prolonged stimuli in previous studies complicate comparisons of psychophysical results, please see lines 529-538. Further, we have discussed how differences between our results, and those of Naarendorp et al., 2010 can be attributed to differences in the spatial properties of the stimulus given that we have both used temporal stimuli that fall within the linear integration range of the mouse rod (Burns et al., 2002) and the critical duration of the human scotopic visual system (Barlow, 1958), see lines 549-556.

8.

Throughout the manuscript (including Figures), it would be informative to provide the flash intensity in photons/μm2/sec (OR photons/m2/s) as well. The figures could report these values across the top x-axis.

This is a good point. We have made the scaling factor that converts photon flux density at the cornea to R*/rod/s explicit in the methods (see lines 310-311) so that one can easily convert between the two sets of units (7 photons/microm/sec at the cornea corresponds to 7R*rod/sec).

9.

Were all mice the same age? If not, they may have different lens densities and so retinal irradiances. Was this considered in the analysis? All mice were approximately the same age, which was ~6 months +/- 2 weeks at the time of the experiment. We have now included this information in lines 80-82.

In our analysis we used the standard of 0.7 for the fraction of photons that are not absorbed by the ocular media, which takes lens density into account (see Lyubarsky et al., 2004), see line 305-308.

10.

I guess the experiments were conducted under binocular viewing (both eyes open). This means the number of photons present in the light can be assumed to be divided in to two eyes because the same photon cannot enter both eyes. How was this accounted for in the computations? By per steradian visual angle calibration, pupil size? The authors also need to consider whether this is automatically accounted for by the visual system with binocular summation. If so, does this affect the estimates of photon absorbed in XX number of rods for absolute threshold? We used the optometer equipped with a 15o lumilens and photometric filter to measure luminace from all surfaces in the behavior chamber. We found that they differed by less than 0.3 log units. This is now explicitly stated in lines 101-104. With this in mind, we measured photon flux density at the location of the mouse in the chamber, at various positions within the chamber, using a radiometric instrument (see point 5 above). In our calculations we assume that this is the photon flux density entering through each pupil of the mouse, as is stated now explicitly in lines 386-389 (also see response to point 12 below). We cannot draw any conclusions concerning the nature of the summation, though it poses an interesting line of future research.

Results:

11.

Do the first two sections fit better in Methods? We have gone back and forth about this ourselves. Several groups have tried to do these experiments and have not been successful, so we consider it an important result that this way of training the mice will converge to a stable threshold. A similar approach to presenting training data in the results section was recently used by Zador et al., 2024 in eLife.

12.

L375: How did the authors come to 1 photoisomerization in every 980 rods? The reader would appreciate the assumptions and calculations involved in this interesting outcome.

We used equation 6 to convert from photon flux density to R*/rod/s. We found the average absolute threshold (defined as the stimulus intensity where d' = 1) to be 0.017 R*/rod/s. This value was multiplied by 0.06 s to arrive at 0.00102 R*/rod. This is equivalent to 1 R* per 980 rods. In multiplying by the flash duration, we assume that the photons are integrated linearly in time. We feel confident in this assumption because 0.06 s is less than the integration time of the mouse rod, and is less than the psychophysically measured human critical duration, as mentioned above. Please see lines 396-389 and the section on determination of retinal irradiance for details (lines 290-311). Note that we now state explicitly that these calculations refer to counts per retina for our full field binocular stimulus.

13.

Given such low number of data points, the ROC fit is not so promising. Could there be an alternative analysis? We agree that having such a low number of data points would make fitting an ROC curve difficult. For this reason, we did not fit the data to the ROC; instead we used the total d' value from all sessions /trials to generate an implied ROC curve. This implied curve was then plotted with the individual points to show that across all 6 mice, day-to-day changes in performance fell along the implied ROC (see lines 486-488). Also, please see response to Reviewer #2, point 19 below, with a similar concern.

Discussion:

14.

The authors assume that false alarm rates in visual psychophysics may arise from variations in stimulus size. This is an interesting thought. Can the authors generate models by combining their data and previous studies to inform the effect of stimulus size and duration on FA rate? This will be a useful addition to psychophysics.

We agree that this is an interesting hypothesis, and that further investigation would be a valuable contribution. However, this is beyond the scope of the current study as our experiments do not address this issue. Our findings, in conjunction with Naarendorp et al., 2010's results, point in this direction. However, without the requisite experiments, we feel that any further analysis would be too speculative.

15.

Naarendorp et al show that the absolute threshold is similar between mice and humans when measured in the peripheral retina. Was this the case in the current study too? This is a good point. We have now added a paragraph comparing our results with a human psychophysical study that most closely approximates our own conditions (Dey et al., 2021) because they used a full-field stimulus. Our thresholds obtained in mice are ~100X higher than those obtained in humans in terms of R*/rod. However, they are only ~6.5X higher when expressed in terms of total R* delivered by the stimulus. It is interesting to note that despite the difference in the size of the eye and total number of rods, the number of R* required for threshold are on the same order magnitude between species. We believe that adding this paragraph adds a new, interesting dimension to our discussion, see lines 570-584.

16.

I think the discussion section too method focused and needs to be expanded on what new we learn with regards to mouse vision and implications in human vision.

We felt that, since we are introducing a new methodology, we should compare with previously used methods and results. With regards to implications about murine and human vision, please see point 15. At the reviewer's suggestion we have re-analyzed our data and found that mouse and human thresholds for full-field stimuli are similar in terms of total R*, despite a 14X difference in the number of rods, see lines 570-584.

Minor comments:

There are several repetitions in the manuscript. For example, 'The signal detection theoretic approach provides estimates of absolute visual threshold' has been stated at least three times. Please minimise such repetitions to make the manuscript more readable.

We have done this, thank you.

Reviewer 2:

In this manuscript the authors are using an elegant 1AFC assay to measure visually guided behavior near detection threshold. Using the theory of signal detection the authors try to understand the impact of response bias near absolute visual threshold. Experiments are elegantly done, and these results will help us understand how decision bias can be a factor in murine psychophysical experiments. It will also have implications for understanding vision near absolute threshold and the major noise components underlying visual detection threshold. There are a few things that could be clarified.

17.

The light stimulus was placed overhead (line 97), indicating stimulus of the ventral retina only. The are significant differences between the dorsal and ventral retina, so this should be clarified. Same goes for the comment on line 535, the light does not seem to cover the whole retina. This could also be taken into account when comparing to previous experiments (Naarendorp 2010, Smeds 2019), where the stimulus was projected to the whole retina.

As indicated in lines 101-104, to the best of our abilities, we made all the surfaces of the chamber equiluminant (within 0.3 log units) to produce uniform illumination of the entire retina, precisely because of this feature of the mouse retina. Please note that the light stimuli used in Naarendorp et al., 2010 and Smeds et al., 2019 were spatially restricted, while our stimulus was designed to be full-field, as described in the discussion, lines 517-529 and 550-557.

18.

The mice were handled under dim red light (line 113) which will affect dark adaptation state. How bright was this light and how could it possibly affect the experiment (pigment bleaching)? This is a good question. The headlamp had a red LED providing < 0.001 scotopic lux at the level of the mouse. By our calculations this amounts to less than 0.2 R*/rod/sec. Handling time for weighing and moving the mouse into the chamber took an average of 1-2 minutes, for a total of 24 R*/rod during this period. Given that 24R* amounts to less 0.001% bleaching of the total rhodopsin in a rod we do not believe that the red lights affected the experiments. We now state the head lamp illumination value in the Methods line118.

19.

For some experiments the conclusions rely on N equal to or less than 3, and bootstrapping is used to get standard errors. The authors should comment on this in more detail, and justify the statistical power of these experiments.

We assume that the reviewer is referring to our ROC experiments (Figures 7 and 8). The goal of these experiments was to determine whether inter-, and intra-, session hit and false alarm rates trend along their implied ROC curve. The power of our analysis comes from the fact that although our pH, pF pairs did not entirely overlap the implied ROC, they did trend along the implied ROC curve. This is remarkable given that the implied ROC is entirely determined by the calculated d' values; there were no free parameters used to plot the ROC, we now comment on this in lines 486-488.

To avoid the limitations imposed by assuming normality of residuals in our regression analysis, we used a non-parametric boot strapping procedure to estimate population parameters such as the standard error and 95% confidence intervals. Our approach only relies on an assumption of binomial sampling, which is standard for analyzing psychometric functions (Kingdom &Prins, 2010). We describe the bootstrapping procedure in lines 318-324.

20.

The non-bias data is elegantly extracted and it seems to be normally distributed, as opposed to Poison. Maybe the authors could elaborate somewhat on the implications of this results, are there more additive noise sources controlling threshold the the noise from photoreceptors, is the discrete noise not controlling absolute visual threshold? We agree with the reviewer and have discussed this issue at length in the discussion, lines 621-638. We added more specific language to include "additive" and "central" noise sources (lines 633-634).

Minor comment: the F in pF is not always subscripted.

Corrected. Thank you! With a few minor clarifications this beautiful work is more than suitable to be published in eNeuro.
